# Associations between saliva and plasma cytokines in cognitively normal, older adults

**DOI:** 10.1007/s40520-022-02292-9

**Published:** 2022-11-01

**Authors:** Georgia M. Parkin, Soyun Kim, Abanoub Mikhail, Rond Malhas, Liv McMillan, Martina Hollearn, Douglas A. Granger, Mark Mapstone, Michael A. Yassa, Elizabeth A. Thomas

**Affiliations:** 1 Department of Epidemiology, University of California Irvine, Irvine, CA USA; 2 Institute for Interdisciplinary Salivary Bioscience Research, University of California Irvine, Irvine, CA USA; 3 Department of Neurobiology and Behavior and Center for the Neurobiology of Learning and Memory, University of California Irvine, Irvine, CA USA; 4 Department of Neurology, School of Medicine, University of California Irvine, Irvine, CA USA; 5 Bloomberg School of Public Health, and School of Medicine, Johns Hopkins University School of Nursing, Baltimore, MD USA

**Keywords:** Biomarker, Cytokine, Saliva, Peripheral, Disease

## Abstract

**Background:**

Inflammatory responses play key roles in the development and progression of many pathological conditions, including neurodegenerative diseases. Accurate quantification of inflammatory factors in saliva would be highly advantageous, given its convenience and non-invasive nature, especially in elderly populations.

**Methods:**

In this study, we measured levels of 10 cytokines, and the pro-inflammatory factor, YKL-40, in plasma and saliva samples from a cohort of nondemented older adults (*n* = 71; 62% female; 70.3 ± 6.4 years) using sensitive electrochemiluminescence-based immunoassays.

**Results:**

We found that the mean levels of all cytokines were higher in saliva compared to plasma and that strong sex differences were observed for both saliva and plasma cytokines in this population. Comparing each cytokine between the two biofluids, we found that levels of interferon-gamma (IFNγ), interleukin (IL)-6 and tumor necrosis factor-alpha (TNFα) in blood were significantly correlated with their respective levels in saliva. We further observed that levels of these cytokines in blood were significantly correlated with additional cytokines in saliva, including IL-1β, IL-10, IL-8, IL12p70 and IL-13.

**Conclusions:**

These findings show that inflammatory markers in saliva are associated with those found in circulation, suggesting shared inflammatory mechanisms between these two fluids. The higher levels of cytokines measured in saliva suggest that it might represent a better peripheral fluid to gauge inflammatory processes. Finally, our findings of robust sex differences in several salivary cytokines could have important implications for their potential use as disease biomarkers in the elderly and might be related to sex differences in the prevalence of age-related conditions.

**Supplementary Information:**

The online version contains supplementary material available at 10.1007/s40520-022-02292-9.

## Introduction

There is a growing number of research studies measuring secreted markers of inflammation, such as cytokines, in peripheral fluids, to better understand the role of inflammation in health and disease. Cytokines are signaling molecules that are released by immune and other (i.e., muscle, adipose) cells, and are major mediators of cellular communication [1]. Cytokines can have pro-inflammatory and/or anti-inflammatory properties and can exhibit autocrine, paracrine or endocrine actions, allowing them to act both locally and distally [2]. Several previous studies have measured levels of cytokines in plasma and serum samples [3–7]. However, one main drawback of measuring cytokine proteins in blood is that endogenous levels of many cytokines are relatively low, with many studies reporting that more than 40% of samples being below the detection limits of the assays for many cytokines [3–11]; This was true even when super-sensitive assays (i.e., Luminex, MSD, Olink^®^, and Simoa) for cytokine quantification were utilized [8, 11, 12].

This issue has also hindered direct comparisons of cytokines in blood to those levels found in saliva with only a handful of studies directly comparing cytokine levels in blood and saliva samples from the same individuals. Perhaps surprisingly, in some studies, cytokines have been reported to be present at higher concentrations in saliva compared to blood [3, 4, 7, 13, 14], suggesting that saliva might represent a better peripheral fluid to gauge inflammatory processes and responses. The non-invasive nature, ease of saliva collection, and ability to collect in any setting further support this idea.

Previous studies that have investigated salivary cytokines did so mostly on adolescent and young adult populations and these studies reported mixed correlations between saliva and blood [3, 5, 6, 14, 15]. However knowledge of the characteristics of saliva and blood cytokines in elderly populations is important given that both innate and adaptive immune responses are affected by the aging process [16]. Additionally, aged individuals tend to present with a chronic low-grade inflammatory state [17, 18] that has been implicated in the pathogenesis of many age-related diseases (atherosclerosis, Alzheimer’s disease, osteoporosis, diabetes, sarcopenia, and chronic inflammatory demyelinating polyneuropathy) [16, 18–23], further suggesting the importance of studying inflammatory markers in aged populations. Only one previous study has reported levels of multiple cytokines in saliva from elderly post-menopausal women [24], but men were not included in the study. Sex-based immunological differences are also known to contribute to variations in the incidence of autoimmune diseases, malignancies and susceptibility to many other diseases, emphasizing that sex is a biological variable should be considered in immunological studies [25].

In this study, to address the general lack of data on cytokine measures in peripheral fluids in the elderly, we measured levels of inflammatory markers in matched blood and saliva samples from a cohort of cognitively normal older adults, using a highly sensitive electrochemiluminescence (ECL)-based, multiplexed immunoassay. Our cohort consisted of both males and females, to examine possible sex differences that might have potential relevance to downstream investigations of inflammation in disease states. We also measured levels of YKL-40, a pro-inflammatory glycoprotein, which has been considered as a pleiotropic cytokine given its secretion by macrophages and involvement in a wide range of inflammatory responses [26, 27] neuroinflammation and astroglial activation [28, 29].

## Methods

### Participants

This study was approved by the University of California, Irvine, Institutional Review Board, in accordance with the requirements of the Code of Federal Regulations on the Protection of Human Subjects. Participants (*n* = 71; 60.5% female; 60–86 years of age) were recruited either from the community-based sample enrolled in the NIA-funded Biomarker Exploration in Aging, Cognition, and Neurodegeneration (BEACoN) Study (R01AG053555Y; *n* = 55) or from the UCI Alzheimer’s Disease Research Center Project 1 (P50AG16573, *n* = 16) (Table 1). Inclusion criteria included being able to speak English fluently, having visual and auditory acuity adequate to complete cognitive assessments, and having normal cognition, defined as a Clinical Dementia Rating of 0 and a Mini-Mental State Examination score of 27 or higher. Participants were excluded if they had a history of significant co-morbid neurology of psychiatric condition, major medical conditions, a diagnosis of MCI or other cognitive impairment, or history of alcohol or substance use disorders within the last two years.

**Table 1:** Summary of participants

	*n*	Mean age (range)	Mean Edu (years)	Mean MMSE (score)	Race (%white)
Males	27	69.3 ± 6.3 years (61–86 years)	16.8	28.2	77.8
Females	44	70.9 ± 6.4 years (60–84 years)	16.0	28.6	84.1
Total	71	70.3 ± 6.4 years (60–86 years)	16.4	28.4	81.7

*Edu* Education, *MMSE* mini-mental state examination

All participants gave written informed consent prior to sample collection, which was done at the baseline visit. Demographic data were collected at the time of sample collection, including sex, age, race, and years of education.

### Plasma collection

Blood samples were obtained from study participants as part of a PET imaging procedure at the UCI Neuroscience Imaging Center (NIC). All participants gave written informed consent for their blood samples to be used for research. Blood was collected without regard to prandial state or medication timing. Prior to the PET scan, blood was drawn via venipuncture from each participant into 3 × 7 mL lavender top EDTA tubes (BD 366450). Immediately after collection, each tube was gently mixed by inverting 8–10 times to ensure proper mixing of blood and anticoagulant, and then placed on wet ice. Blood samples were centrifuged in a swinging rotor bucket within 1 h of collection at 2600 × RPM at 20 °C for 10 min. The isolated plasma was transferred and pooled into a sterile 50 mL polypropylene conical tube and mixed by inversion a few times. The plasma samples were aliquoted by 0.750 mL increments into 2 mL polypropylene cryovials. The plasma aliquots were transferred into a − 80 °C freezer for storage until required for analyses. Forty-five subjects who provided a plasma sample also provided a saliva sample; however, only 23 of these subjects provided both samples on the same day. All samples were collected between the hours of 10 am and 4 pm.

### Saliva collection

All donors were asked to refrain from smoking, eating, drinking, or oral hygiene procedures for at least 1 h prior to samples collection. Saliva samples were collected between 10 am and 4 pm using the passive drool method according to the previously established protocols [30]. Roughly two milliliters of unstimulated whole saliva was obtained. Besides the subjects who provided a plasma sample, some subjects provided a saliva-only sample. Samples were immediately frozen at − 20C at the time of collection, then stored at − 80C. At the time of use, saliva samples were thawed and centrifuged (10,000 g; 10 min; 4C) to remove mucins, insoluble material and cellular debris. Supernatants were collected and used for all assays.

### Determination of cytokine levels

Cytokine levels in saliva and blood were measured in duplicate using the V-PLEX Proinflammatory Cytokine Panel 1 10-plex ECL immunoassay (Meso Scale Discovery (MSD), Gaithersburg, MD). ECL-based immunoassays are superior to traditional immunoassays (i.e., ELISA), by having greater dynamic ranges and greater sensitivity of detection. For plasma, we used 50 μl volume diluted 1:2 in Diluent 2 (MSD). Assays were run according to MSD manufacturers protocol, but with two additional, lower standard curve points. Because this assay was validated for use on plasma/serum samples, we investigated the optimal dilution for use in saliva, which was the same as plasma at 1:2. Because previous studies indicated low recovery of multiplexed cytokines in the saliva matrix [4], we determined recovery of each cytokine in the saliva matrix by calculating the recovery of a spiked-in known standard in saliva samples from *n* = 11 control individuals. We found that all cytokines, with the exception of IL-4, showed mean recoveries in saliva between 85.7 and 105.2% (Suppl. Table 1). These are consistent with the reported recoveries in EDTA plasma, according to the manufacturer (Suppl. Table 1). Plasma and saliva samples from the same individual were, therefore, run on the same V-PLEX plate, to optimize comparisons between these two fluids. Cytokine concentrations (pg/ml) were determined with MSD Discovery Workbench Software using curve fit models. Lower limits of detection (LLoD) were calculated as the concentration corresponding to the signal 2.5 times standard deviation above background. LLoDs and intraassay CVs determined are as follows: IFNγ (0.028 pg/ml, 4.4%), IL-1β (0.017 pg/ml, 5.5%), IL-2 (0.012 pg/ml, 5.1%), IL-4 (0.005 pg/ml, 8.9%) IL-6 (0.03 pg/ml, 5.3%), IL-8 (0.018 pg/ml, 6.4%), IL-10 (0.011 pg/ml, 5.1%), IL-12p70 (0.011 pg/ml, 16.3%), IL-13 (0.03 pg/ml, 10.5%) and TNFα (0.038 pg/ml, 8.9%). YKL-40 concentrations were measured using the U-PLEX YKL-40 human assay (MSD, Gaithersburg, MD; Cat #:K151VLK; LLD, 0.061 pg/ml, 5.2%). For this assay, saliva and blood samples were diluted 1:1,000 in Diluent 43 prior to use in the assay. Spike and recovery assays were carried out for YKL-40 in saliva as above and was found to be 101.4% (Suppl. Table 1).

### Analytic strategy

Raw data were first tested for normality using Shapiro–Wilk and Kolmogorov–Smirnov normality tests. All raw data from both fluids were not normally distributed; therefore, initial comparison of cytokine levels with age and sex were carried out using Spearman correlation analysis and Mann–Whitney *U* test (GraphPad Prism). Outliers were determined using Iglewicz and Hoaglin with an absolute Z score threshold of 5, resulting in the removal of 1 value for each IL-6, IL-8 and TNFα in blood samples only. The intercorrelations between and among plasma and salivary cytokines were carried out on log-transformed data using partial correlations, controlling for age and sex (IBM^®^ SPSS^®^ Statistics version 25 for Windows; BM Corp., NY, USA). A Bonferroni correction was employed for all linear correlations to correct for multiple tests. For initial analyses, values below detection were omitted. In secondary analyses, values below the detection limit were replaced with the LLoD, but only when < 30% of the samples were below the detection limit.

## Results

### Descriptive characteristics for salivary and plasma cytokine levels

Proinflammatory markers (TNFα, IL1β, IL-2, IL-6, IL-8, IL-12p70, IFNγ, and YKL-40) and anti-inflammatory markers (IL-4, IL-10, and IL-13) were measured in plasma and saliva samples from a cohort of cognitively normal elderly participants (*n* = 71 in total, see Table 1). Descriptive statistics for these factors are presented in Table 2. The median levels of salivary cytokines were higher than the corresponding median level in plasma, with the exception of IFNγ (Table 2). Consistent with prior studies, many cytokines measured in blood, including IL-12p70, IL-13, IL-1β, IL-2, and IL-4, were below the lower limit of detection for > 30% of the samples (40.8–71.4% of samples; Table 3). Therefore, these plasma cytokines were not included in additional analyses. In contrast, for saliva, only IL-12p70 showed high undetected levels, with 25.4% of samples returning below detection levels for this cytokine (Table 3). Considering only the subset of *n* = 23 subjects with exact day matching of sample collection, no subject had above detection levels for all cytokines and YKL-40 in both saliva and blood, suggesting that day of collection was not a factor in the detection rates of analytes. Notably, levels of YKL-40 were a magnitude more abundant (ng/ml compared to pg/ml) in both biofluids compared to the other cytokines (Table 2).

**Table 2:** Summary of cytokine data in saliva and plasma samples

	*n*	Mean ± S.D. pg/ml	Median	Range
Saliva				
IFN-γ	54	15.49 ± 66.7	0.819	(0.028–396.3)
IL-10	58	2.67 ± 8.07	0.456	(0.021–50.2)
IL-12p70	42	0.219 ± 0.421	0.044	(0.012–1.81)
IL-13	59	7.02 ± 6.12	4.421	(0.589–31.59)
IL-1ß	62	259.2 ± 365.9	148.3	(7.08–1912)
IL-2	49	1.27 ± 5.50	0.154	(0.014–38.21)
IL-4	41	0.093 ± 0.178	0.025	(0.006–0.885)
IL-6	66	13.39 ± 37.50	2.56	(0.057–238)
IL-8	59	1567.0 ± 924.5	1438.0	(175.5–3415)
TNF-α	59	10.90 ± 22.9	4.25	(0.537–149.2)
YKL-40	57	49,665 ± 90,775	20,566	(1917–585,207)
Plasma				
IFN-γ	49	3.77 ± 5.83	1.97	(0.852–37.37)
IL-10	44	0.082 ± 0.079	0.054	(0.011–0.355)
IL-12p70	14	0.062 ± 0.104	0.024	(0.013–0.407)
IL-13	21	0.360 ± 0.341	0.206	(0.035–1.238)
IL-1ß	8	0.191 ± 0.379	0.021	(0.019–0.870)
IL-2	16	0.036 ± 0.046	0.021	(0.012–0.205)
IL-4	4	0.061 ± 0.107	0.008	(0.005–0.222)
IL-6	47	4.96 ± 30.9	0.277	(0.03–212.3)
IL-8	49	3.57 ± 2.85	3.196	(0.909–21.1)
TNF-α	48	1.25 ± 1.36	0.921	(0.399–9.93)
YKL-40	49	26,479 ± 29,424	16,058	(3529–18,0121)

Mean and median values shown include only those values that were above the lower limit of detection

**Table 3:** Detection rates of cytokines in saliva and plasma

	Number of samples below detection	
	Saliva *n*, (% of total)	Plasma *n*, (% of total)
IFN-γ	5 (8.4%)	0 (0%)
IL-10	1 (1.6%)	5 (10.2%)
IL-12p70	17 (28.8)	28 (57.1%)
IL-13	0 (0%)	30 (61.2%)
IL-1β	0 (0%)	41 (83.4%)
IL-2	10 (16.9%)	33 (67.3%)
IL-4	18 (30.0%)	45 (91.8%)
IL-6	0 (0%)	2 (4.1%)
IL-8	0 (0%)	0 (0%)
TNF-α	0 (0%)	1 (2.0%)
YKL-40	2 (3.3%)	0 (0%)

*IFNγ* Interferon-gamma, *IL* interleukin, *TNFα* tumor necrosis factor-alpha

### Sex and age effects of salivary and plasma cytokine levels

**Fig. 1:**
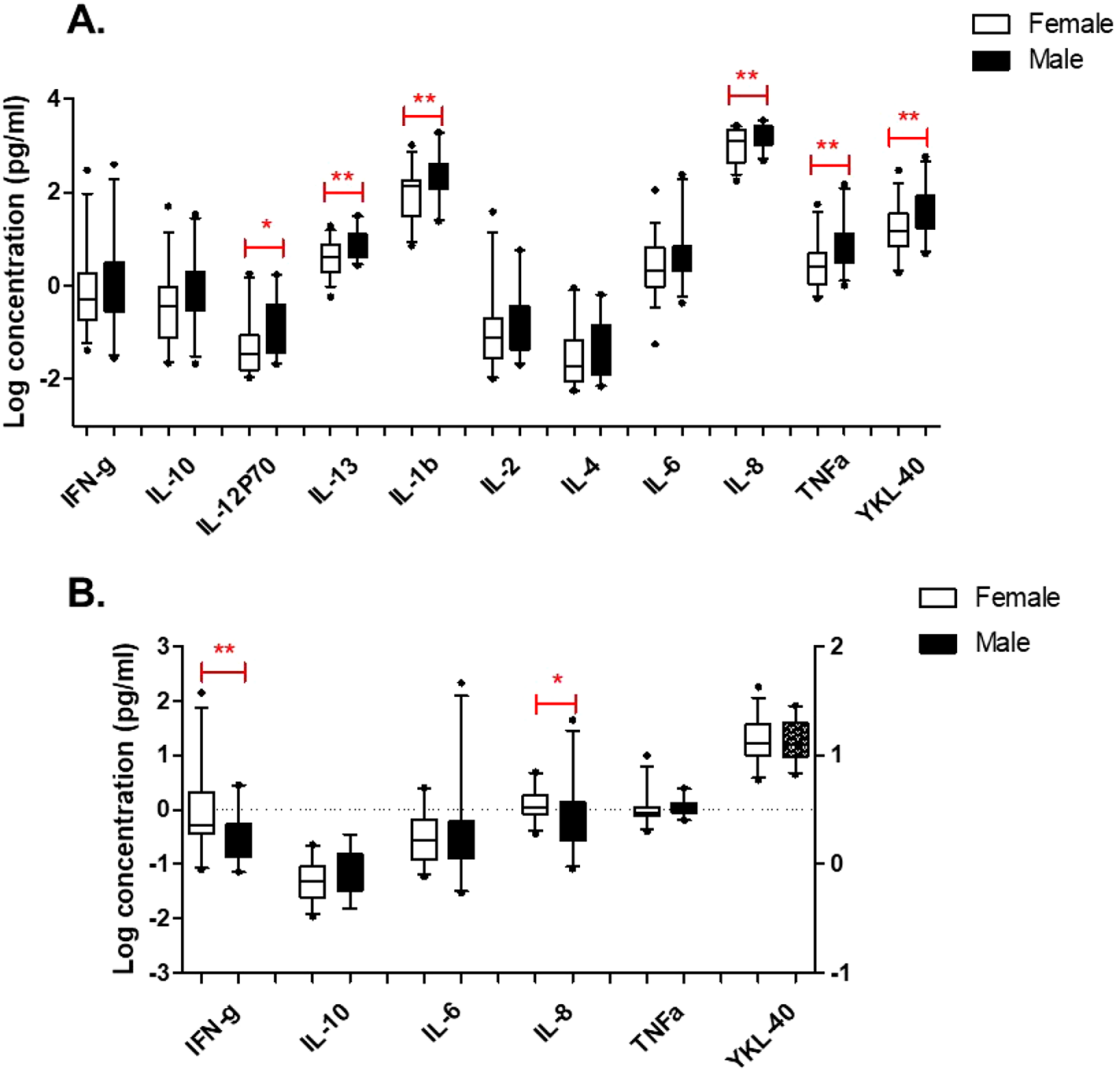
Sex effects of salivary (**A**) and plasma (**B**) cytokine levels. Proinflammatory markers (TNFα, IL1β, IL-2, IL-6, IL-8, IL-12p70, IFNγ, and YKL-40) and anti-inflammatory markers (IL-4, IL-10, and IL-13) were measured in plasma and saliva samples from a cohort of cognitively normal elderly participants (*n* = 71; 62% female; mean age 70.3 ± 6.4 years). Plasma levels of IL-1b, IL-2, IL-4, IL-12p70, and IL-13 were below detection in > 30% of samples, hence were not included in the analyses. Significant differences were determined using Student’s *t* test on Log-transformed values: *p < 0.05; **p < 0.01

There were no significant correlations between any salivary cytokines and age, although there was a narrow age range of this cohort (60–86 years). In contrast, there were modest significant correlations between age and blood levels of IL-2 (*r* = 0.517; *p* = 0.039) and IL-6 (*r* = 0.315; *p* = 0.033), despite the narrow age range of the participants. With regards to sex effects, interestingly, significant differences were observed for IL-12p70, IL-1β, IL-13, IL-2, IL-8, TNF-α and YKL-40, with these analytes showing higher levels in males compared to females (Fig. 1A). Similar results were obtained when values below the LLoD were replaced with the LLoD, which was only done for analytes that had < 30% of samples below the LLoD value (data not shown). In contrast, plasma levels of IFN-γ and IL-8 were higher in females compared to males (Fig. 1B). No other sex differences were found with any cytokines or YKL-40 in blood.

### Intercorrelations among inflammatory factors

Next, we performed intercorrelation analyses among cytokines and YKL-40, using partial correlation analyses correcting for age and sex, in light of the effects observed above. All of the salivary cytokines, as well as YKL-40, were strongly, positively intercorrelated, with rho values ranging from *r* = 0.397 to as high as *r* = 0.789 (all *p* values < 0.001; Suppl Table 2). Salivary TNFα showed the most significant correlations to other salivary cytokines (Suppl Table 2). Cytokines measured in plasma were also significantly correlated with one another, but to a lesser extent compared with saliva (Suppl Table 3). Plasma TNFα levels showed the highest correlations to plasma IFNγ (*r* = 0.517; *p* < 0.001) and IL-10 (*r* = 0.482; *p* < 0.001). Levels of YKL-40 in plasma were only correlated with plasma levels of TNFα (*r* = 0.417; *p* = 0.004) (Suppl Table 3).

### Saliva-plasma associations

Next, we tested whether individual levels of each cytokine and YKL-40 were correlated in saliva and blood. Only those analytes that were above the LLoD > 30% of samples for any cytokine were included, leaving IL-6, IL-8, IL-10, IFNγ, TNFα a and YKL-40 for comparisons between both fluids. Partial correlation analysis, correcting for age and sex revealed statistically significant correlations for IFNγ, IL-6 and TNFα in blood with their corresponding level in saliva (Table 4). Unadjusted correlations and correlation analyses using replaced values for those < LLoD revealed similar results (Data not shown). When comparing a given analyte in plasma to all other analytes in saliva, we found additional significant correlations (Table 5). For example, blood levels of IFNγ, IL-6, and TNFα were significantly correlated with other cytokines in saliva. Notably, plasma IFNγ was significantly correlated with all salivary cytokines except, IL-6 and IL1β prior to Bonferroni multiple test correction and significantly correlated with IL-10 and TNFα after correction (Table 5). Similarly, plasma levels of IL-6 were significantly correlated with all other salivary cytokines except IL-13 prior to Bonferroni correction, and significantly correlated with IL-10, IL-1β and IL-2 after correction (Table 5). Salivary levels of YKL-40 were robustly significantly correlated with plasma levels of IL-6 and TNFα (Table 5). Analyses were repeated with values below the LLoDs, and similar results were obtained but with lower rho and *p* values, despite the larger sample size (Data not shown).

**Table 4:** Correlations of individual cytokines between saliva and blood

Cytokine	Rho	*P* value
IFNγ	**0.370**	**0.022**
IL-10	0.161	0.335
IL-6	**0.359**	**0.023**
IL-8	− 0.104	0.508
TNFα	**0.386**	**0.012**
YKL40	0.026	0.871

Partial correlation analysis was carried out correcting for age and sex. Significant correlations are indicated in bold font

*IFNγ* Interferon-gamma, *IL* interleukin, *TNFα* tumor necrosis factor-alpha

**Table 5:** Correlations between plasma and salivary cytokines

	PLASMA					
	IFNγ rho, *p* value	IL-10 rho, *p* value	IL-6 rho, *p* value	IL-8 rho, *p* value	TNFα rho, *p* value	YKL-40 rho, *p* value
SALIVA						
IFNγ	**0.37, 0.022**	0.166, 0.348	**0.411, 0.014**	0.107, 0.524	0.315, 0.058	0.077, 0.647
IL-10	*0.547, < 0.001	0.161, 0.335	*0.423, 0.007	0.07, 0.659	**0.381, 0.014**	0.051, 0.749
IL-6	0.225, 0.147	0.045, 0.790	**0.359, 0.023**	0.137, 0.382	0.13, 0.413	− 0.007, 0.964
IL-8	0.294, 0.056	0.066, 0.694	**0.336, 0.034**	− 0.104, 0.508	0.12, 0.449	− 0.129, 0.409
TNFα	*0.478, < 0.001	0.163, 0.33	**0.339, 0.032**	0.055, 0.725	**0.386, 0.012**	− 0.148, 0.345
IL-12p70	**0.44, 0.015**	0.274, 0.175	**0.405, 0.036**	0.103, 0.587	*0.516, 0.004	0.089, 0.641
IL-13	**0.301, 0.05**	− 0.076, 0.650	0.134, 0.408	− 0.043, 0.784	0.264, 0.091	− 0.130, 0.406
IL-1β	0.179, 0.251	0.198, 0.233	*0.425, 0.006	0.05, 0.752	0.25, 0.110	− 0.112, 0.476
IL-2	**0.389, 0.021**	0.209, 0.251	*0.48, 0.005	− 0.037, 0.834	0.277, 0.113	0.218, 0.208
IL-4	**0.387, 0.046**	0.031, 0.884	**0.486, 0.016**	0.288, 0.146	0.371, 0.062	0.223, 0.263
YKL-40	0.274, 0.083	0.203, 0.227	**0.384, 0.017**	0.008, 0.958	*0.481, 0.002	0.019, 0.911

Partial correlations between plasma and salivary cytokines, corrected for age and sex

Plasma levels of IL-1β, IL-2, IL-4, IL-12p70 and IL-13 were below detection in > 30% of samples, hence were not included in the analyses

*IFNγ* Interferon-gamma, *IL* interleukin, *TNFα* tumor necrosis factor-alpha

Uncorrected *p* values < 0.05 are highlighted in bold. Bonferroni-adjusted *p* values (*p* < 0.0083) are indicated by asterisks

## Discussion

In this study, we measured a panel of 10 cytokines and the pro-inflammatory factor, YKL-40, in saliva and plasma samples from a cohort of nondemented older adults to catalog levels of these analytes in older subjects. Similar to previous studies, we found that salivary levels of most cytokines were higher than those found in plasma, and that blood levels for IL-12p70, IL-1β, IL-2, IL-4, and IL-13 were below the detection levels in a majority of samples tested [3, 4, 13–15]. Although at least one previous study reported higher levels of several cytokines in serum compared to saliva, different methods were used to measure cytokines in the two fluids, making direct comparisons across these two fluids difficult [8, 31]. In our study, blood and saliva samples from the same subject were measured on the same plate, to ensure the most reliable comparisons possible.

Comparing cytokines to one another within each biofluid, we found strong intercorrelations of cytokine concentrations. This is likely due to the fact that cytokines are known to be produced in a cascade, whereby one cytokine stimulates the production and secretion of additional cytokines [2]. Such correlations among salivary cytokines have also been reported in other studies [31]. In addition to correlations detected among pro-inflammatory cytokines, we also observed significant positive correlations between pro-inflammatory (i.e., TNFα) and anti-inflammatory (i.e., IL-10) cytokines. Anti-inflammatory cytokines are released in response to elevated levels of pro-inflammatory cytokines, in attempts to counter-regulate production and function of these cytokines to limit an inflammatory cascade. Among all the anti-inflammatory cytokines, IL-10 has one of the most potent anti-inflammatory properties and is known to repress the expression of TNFα, IL-6 and IL-1 by activated macrophages [2]. In light of this known effect, it is not surprising that levels of these opposing cytokines might be correlated. Alternatively, some cytokines have both pro- and anti-inflammatory properties, depending on the context, which could account for some of the intercorrelations.

We also investigated YKL-40, a secreted glycoprotein and pleiotropic cytokine, which is expressed in a wide variety of tissues [32] and is involved in a wide range of inflammatory responses [26, 27]. We found that YKL-40 in both plasma and saliva was highly correlated with cytokine levels within each fluid. While levels of YKL-40 were not significantly correlated between blood and saliva, we did observe that salivary YKL-40 was significantly correlated with blood levels of IFNγ, IL-6 and TNFα. These findings are consistent with previous studies demonstrating upregulation of YKL-40 in a number of inflammatory conditions, in response to the pro-inflammatory cytokines TNFα and IL-1β [33, 34].

Comparing concentrations of individual cytokines between the two peripheral biofluids, we found significant correlations between saliva and plasma for IFNγ, IL-6 and TNFα. IFNγ and IL-6 are the cytokines most consistently reported as showing significant correlation between saliva and blood [3, 6, 13, 24, 35]. Our own previous studies found a significant correlation between plasma and saliva for IL-6 (*r* = 0.590; *p* < 0.0001) in adult patients with Huntington’s disease and normal control subjects (aged 23–78 years) [35]. Other studies have found more modest correlations between saliva and blood for IL-6 in older women (*r* = 0.29; *p* = 0.02) [24] and in healthy adults (*r* = 0.31; *p* < 0.01) [6]. And finally, some studies have not reported significant correlations between fluids for IL-6 [3, 5, 6, 36]. Although in one study, serum and salivary IL-6 levels were compared in response to exercise, whereby differences between the systemic/muscular and the salivary routes of IL-6 production might be related to the lack of correlation [37]. Other studies have found significant correlations for IFNγ between saliva and serum [3, 6], but no correlations for a range of other cytokines [5, 6, 36]. When non-detectable levels were included as zero in correlations analyses, salivary levels of IL-2, IL-12p70 and interferon IFNγ correlated with their serum counterparts [3] and a positive correlation between saliva and serum levels for IL-1β in healthy adolescent girls was observed [5]. Although, it should be noted that caution must be used when interpreting findings from data where values below detection have been replaced by zero or the LLoD in > 30% of samples.

The assessment of systemic cytokines is challenging given that these factors are typically present at very low levels in blood samples and, hence, require highly sensitive technologies for their detection. However, past studies using super-sensitive assays have still reported below detection levels for > 40% of blood samples measuring IFNγ, IL-4 and IL-13 (Luminex platform [8]) and > 50% for IL-2, IL-4, IL13, TNFα and IFNγ (Olink^®^ platform [9]). A recent study compared cytokine quantification across five leading platform technologies focusing on the most common cytokines, IL-1β, IL-6, TNF-α, and IFN-γ [11]. This paper found that the assay with the highest sensitivity in detecting endogenous analytes across all analytes and clinical populations was the Simoa^™^ platform. However, in this study, the Simoa platform still reported 40% of samples were below detection for IL1β, 50% for IFNγ and > 80% for IL2 and IL4 in plasma samples from healthy control subjects, with much higher rates of samples below detection for the other platforms [11]. Another study reported similar findings [12]. Additionally, it has been shown that differences between the use of serum vs. plasma (i.e., the presence of blood clotting factors in serum) may differentially affect measurements of inflammatory markers as shown in previous studies [11], which is something that should be considered in future studies.

While our studies, and others, would suggest that saliva is not a robust surrogate for blood with respect to the identical cytokine tested, we further tested for associations between different cytokines and found that blood levels of several cytokines were significantly associated with salivary levels of others. For example, plasma levels of IFNγ were highly correlated with levels of TNFα, IL-12p70, IL-2 and IL-10 in saliva. Similarly, plasma levels of IL-6 were significantly correlated, to various extents, with all other salivary cytokines except IL-13 and salivary levels of YKL-40 were significantly correlated with plasma levels of IL-6 and TNFα. These findings indicate that cytokine levels are indeed related between saliva and blood, and suggest that inflammatory mechanisms are shared, at least to some extent, across peripheral fluids.

It may not be surprising that some cytokines are related between blood and saliva. Constituents from the blood can enter into the saliva via several mechanisms, including transcellular transport, passive intracellular diffusion or active transport. Also, cytokines in saliva might come from serum-derived cytokines infiltrating into the mouth via crevicular fluid, oral injuries, or other tissue damage [38]. However, cytokines are also known to be produced locally by buccal cells, gingival epithelial cells, or resident immune cells. Cytokines might be released from the salivary glands, which are innervated by parasympathetic and sympathetic efferent nerves. These multiple sources of salivary cytokines could be one reason for low correlations observed between saliva and blood.

With respect to the latter source, the contribution of neutrophils to salivary cytokines might be considerable, especially under pathological conditions, where recruitment of neutrophils into the oral cavity is accelerated. Another reason for poor correlations between fluids for some cytokines is the presence of local inflammation, resulting from poor oral hygiene, gingivitis, periodontal disease, or oral cancer [39–42]. Under such conditions, recruitment of neutrophils into the oral cavity is accelerated, which can result in a higher local production of inflammatory factors. In particular, IL1β and IL-6 have shown to be elevated in subjects with periodontal disease [43], although both pro-inflammatory and anti-inflammatory cytokines have also been implicated in periodontal disease [44]. However, it should be noted that not all studies found similar associations [45]. One limitation of the current study is that oral health indicators were not collected on these subjects. Dental and oral diseases remain problematic for many Americans. According to the Centers for Disease Control and Prevention (CDC), 47% of US adults 30 years of age or older have periodontal disease [46]. There is a growing interest in the role of oral health in systemic disease incidence and prevention, hence investigating the roles of oral health on peripheral cytokines measures is warranted. Finally, studies have suggested increased contamination of plasma components in saliva samples from elderly individuals [47], which could also affect salivary levels of analytes.

Another potentially important result from our study is the robust sex effect we observed in both saliva and plasma cytokines. We found that levels of IL-12p70, IL-1b, IL-13, IL-2, IL-8, TNFα, and YKL-40 were all higher in males compared to females. In contrast, plasma cytokines did not show greater levels in males, except for IFNγ and IL-8, where levels were higher in females compared to males. Other studies on younger adult populations and adolescents did not observe similar differences between males and females [48, 49]; hence, this finding might represent a state that is unique to older populations. With age, concentrations of sex steroids decline rapidly for females and more gradually for males, paralleling a progressive functional decline in the immune system of both sexes [16]. Many cytokines, including IL-1β, IL-2, IL-6, and TNFα, are regulated by the female sex hormone, estrogen, which generally promotes inflammation at low levels and dampens inflammation at high levels [25, 50]. Our cohort of elderly women (aged 60–86 years) would be expected to be post-menopausal, which might explain the increases in plasma levels of IFNγ and IL-8, the latter of which specifically been shown to have a negative correlation with serum estradiol [50].

The increases in salivary cytokines observed in men compared to women might be related to the fact that men exhibit greater comorbidities than women in aged populations. Chronic inflammation has been implicated in the pathogenesis of many age-related diseases, including atherosclerosis, Alzheimer’s disease, osteoporosis, and diabetes. Nonetheless, these sex differences in aged population have implications for the use of peripheral cytokines as biomarkers for a range of conditions.

## Conclusions

Growing evidence suggests that high levels of pro-inflammatory markers in older people are associated with risk of developing age-related diseases, as well as multiple chronic diseases [17, 51], although the specific mechanisms that connect inflammation with chronic diseases remain unclear. Our studies suggest that inflammatory markers in saliva and blood are indeed related, and that sex differences exist for both saliva and plasma cytokines. Additional research and progress in this field are greatly needed, if inflammatory pathways might be targeted as interventions that promote healthy and successful aging.

## Supplementary Information

Below is the link to the electronic supplementary material.

Supplementary file1 (DOCX 32 KB)
