# Supplementary material for: Associations between saliva and plasma cytokines in cognitively normal, older adults
Source: Aging Clin Exp Res. 2022 Nov 1;35(1):117–26. doi: 10.1007/s40520-022-02292-9 (PMC9816252; doi:10.1007/s40520-022-02292-9)
Supplement: Supplementary file 1 — Supplementary file1 (DOCX 32 KB) [file 40520_2022_2292_MOESM1_ESM.docx]

Suppl Table 1. Recovery rates for cytokines in saliva and plasma.

Recovery of each analyte in the saliva matrix was determined by calculating the recovery of a spiked-in known standard in saliva samples from n=11 control individuals. Interferon-gamma (IFNγ), interleukin (IL), and tumor necrosis factor-alpha (TNFα).

Suppl Table 2. Partial correlations among saliva cytokines, corrected for age and sex.

The intercorrelations among salivary cytokines were carried out on log-transformed data using partial correlations, controlling for age and sex (IBM SPSS Software). The Bonferroni corrected p-value = 0.0045 and are highlighted in bold font.

Suppl Table 3. Partial Correlations of plasma cytokines, correcting for age and sex.

The intercorrelations among plasma cytokines were carried out on log-transformed data using partial correlations, controlling for age and sex (IBM SPSS Software). Only those analytes that were detectable in >70% of samples were used. The Bonferroni corrected p-value = 0.008 and are highlighted in bold font.
